# The microtubule-associated histone methyltransferase SET8, facilitated by transcription factor LSF, methylates α-tubulin

**DOI:** 10.1074/jbc.RA119.010951

**Published:** 2020-02-28

**Authors:** Hang Gyeong Chin, Pierre-Olivier Esteve, Cristian Ruse, Jiyoung Lee, Scott E. Schaus, Sriharsa Pradhan, Ulla Hansen

**Affiliations:** ‡ New England Biolabs, Ipswich, Massachusetts 01938; § MCBB Graduate Program, Graduate School of Arts and Sciences, Boston University, Boston, Massachusetts 02215; ¶ Biological Resource Center, Korea Research Institute of Bioscience and Biotechnology, Ipsin-gil, Jeongeup-si, Jeollabuk-do 56212, South Korea; ‖ Center for Molecular Discovery, Boston University, Boston, Massachusetts 02215; ** Department of Biology, Boston University, Boston, Massachusetts 02215

**Keywords:** tubulin, posttranslational modification (PTM), protein methylation, cytoskeleton, cancer, lysine methyltransferase 5A (KMT5A), mammalian cells, SET8, transcription factor CP2 (TFCP2), transcription factor LSF

## Abstract

Microtubules are cytoskeletal structures critical for mitosis, cell motility, and protein and organelle transport and are a validated target for anticancer drugs. However, how tubulins are regulated and recruited to support these distinct cellular processes is incompletely understood. Posttranslational modifications of tubulins are proposed to regulate microtubule function and dynamics. Although many of these modifications have been investigated, only one prior study reports tubulin methylation and an enzyme responsible for this methylation. Here we used *in vitro* radiolabeling, MS, and immunoblotting approaches to monitor protein methylation and immunoprecipitation, immunofluorescence, and pulldown approaches to measure protein–protein interactions. We demonstrate that *N*-lysine methyltransferase 5A (KMT5A or SET8/PR-Set7), which methylates lysine 20 in histone H4, bound α-tubulin and methylated it at a specific lysine residue, Lys^311^. Furthermore, late SV40 factor (LSF)/CP2, a known transcription factor, bound both α-tubulin and SET8 and enhanced SET8-mediated α-tubulin methylation *in vitro*. In addition, we found that the ability of LSF to facilitate this methylation is countered by factor quinolinone inhibitor 1 (FQI1), a specific small-molecule inhibitor of LSF. These findings suggest the general model that microtubule-associated proteins, including transcription factors, recruit or stimulate protein-modifying enzymes to target tubulins. Moreover, our results point to dual functions for SET8 and LSF not only in chromatin regulation but also in cytoskeletal modification.

## Introduction

Microtubules (MTs),^4^ the polymerized heterodimers of α-tubulin and β-tubulin, are major cytoskeletal components that play important roles in key cellular processes such as structural support, localization of organelles, and chromosome segregation (1, 2). A number of posttranslational modifications (PTMs) of tubulins have been reported that contribute to the functional diversity of MTs and affect MT dynamics and organization (3). This led to the hypothesis of a tubulin code (1), in which tubulin modifications specify biological outcomes through changes in higher-order microtubule structure by recruiting and interacting with effector proteins. As in the well-established, parallel histone code paradigm, each specific modification would be anticipated to directly recruit or interrupt the interaction between MTs and specific interactor(s). Most identified tubulin PTMs, including tyrosination, glutamylation, and glycylation, map to the unstructured tubulin C termini that regulate interaction with motors and other microtubule-associated proteins (MAPs) (3). The extensively studied acetylation on Lys^40^ of α-tubulin is unusual in that it is located in the lumen of MTs (4); this modification marks stable MTs and may be induced by transient breakage (5, 6). Notably, tubulin methylation has been less studied than other types of tubulin modification.

SET8/PR-Set7 is an *N*-lysine methyltransferase responsible for monomethylation of histone and nonhistone proteins in higher eukaryotes (7). It is functionally characterized as a histone H4 lysine 20–specific monomethyltransferase (8); this modification is often a mark for transcriptional repression, although it can also be associated with active promoters. SET8 and H4K20me are specifically enriched during mitosis (9, 10). SET8 is required for DNA replication and mitosis during cell cycle progression, with deletion or RNAi-mediated depletion of the enzyme leading to impaired replication origin licensing and reduced chromosome compaction (11–18). Previous findings, in particular, suggested that SET8 and H4K20me1 are required for mitotic entry (19). In addition, enhanced expression or impaired cell cycle–specific degradation of SET8 can lead to premature chromosome condensation, mitotic delay, or impaired cytokinesis (20, 21). SET8 also mediates monomethylation of other substrates, including p53, which results in repression of p53 target genes (22). However, how H4K20me1 is regulated and how it functions to promote cell cycle progression remains an open question, including the possibility that other nonhistone substrates may be involved.

LSF (also named CP2), previously characterized widely as a transcription factor, is an oncogene in hepatocellular carcinoma that is significantly overexpressed in hepatocellular carcinoma cell lines and patient samples (23–28) as well as in other cancer types (29). LSF is involved in cell cycle progression and cell survival (30–32). Initially, LSF was described as a regulator of G_1_/S progression (32) and as essential for inducing expression of the gene encoding thymidylate synthase (*TYMS*) in late G_1_. Additional involvement of LSF in mitosis was initially demonstrated through characterization of the effects of factor quinolinone inhibitor 1 (FQI1), a specific small-molecule inhibitor of LSF (31). The biological specificity of FQI1 for LSF was confirmed by the parallel mitotic phenotypes between treatment with FQI1 and with siRNA targeting LSF (33). These include mitotic delay with condensed but unaligned chromosomes, incomplete cytokinesis, and multinucleation. FQI1 not only abrogates the DNA-binding and corresponding transcriptional activities of LSF (31) but also specific LSF–protein interactions (34). Finally, FQI1 inhibits growth of hepatocellular carcinoma tumors in multiple mouse models and causes cell death via mitotic defects in hepatocellular carcinoma cell lines (31, 35).

In this study, we demonstrate that these three regulators of mitosis, SET8, LSF, and α-tubulin, interact with each other *in vitro* and within cells. Furthermore, we demonstrate that SET8 is a microtubule-associated methyltransferase that specifically methylates Lys^311^ of α-tubulin *in vitro*. Finally, in parallel to how transcription factors stimulate histone modification by interacting with chromatin writers and DNA, LSF stimulates *in vitro* methylation of α-tubulin by SET8. Overall, these results suggest that LSF and SET8 have biological implications beyond gene transcription and histone methylation, respectively.

## Results

### SET8 interacts directly with α-tubulin

**Figure 1.**
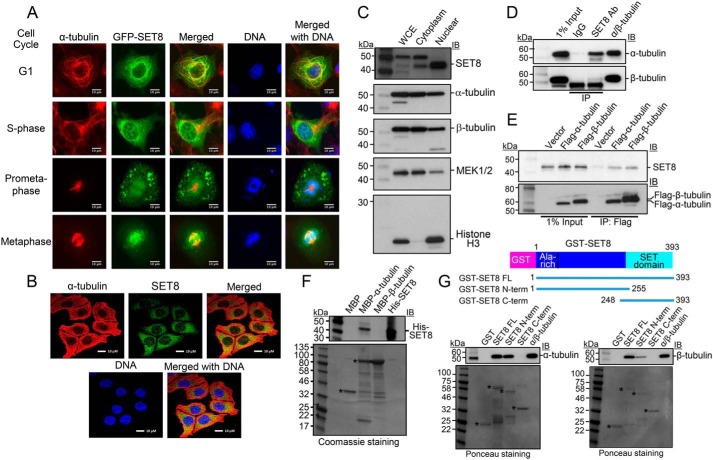
**SET8 associates with tubulin in cells and directly interacts with α-tubulin *in vitro*.** *A*, colocalization of SET8 and α-tubulin in COS7 cells. GFP-SET8 (*green*) was expressed in asynchronous cells, tubulin was detected with anti-α-tubulin antibody (*red*), and DNA with DAPI (*blue*). *Yellow* in the merged image indicates colocalization of SET8 and α-tubulin. Images are from cells identified as being in the indicated stages of cell cycle progression. *Scale bars* = 10 μm. *B*, colocalization of endogenous SET8 and α-tubulin in human HCT116 cells. *C*, endogenous SET8 is localized in the nucleus and cytoplasm in HEK293T cells. 10 μg each of whole-cell extract (*WCE*), cytoplasmic, and nuclear fractions were analyzed for the presence of SET8, α- and β-tubulins (cytoplasmic marker), MEK1/2 (predominantly cytoplasmic marker), and histone H3 (nuclear marker). *D*, coimmunoprecipitation from HEK293T cells of endogenous tubulins with endogenous SET8, using SET8 antibody (Ab). *Right lane*, more than 99% pure tubulin (MP Biomedicals, 08771121) as a positive control. Immunoprecipitates were analyzed using antibodies against the indicated proteins by immunoblot (*IB*). *E*, coimmunoprecipitation from HEK293T cells of endogenous SET8 with transiently expressed FLAG-tagged tubulins, as detected by immunoprecipitation (*IP*) with antibody against FLAG. *F*, MBP pulldown analysis of purified His-SET8 with MBP–α-tubulin but not MBP–β-tubulin. *Top*, IB in which biotinylated molecular mass markers are visualized. *Bottom*, Coomassie staining of the same gel (shown in *grayscale*) in which standard molecular mass markers are visualized. *Asterisks*, expected positions of migration of the MBPs. *G*, GST pulldown analysis of purified porcine brain tubulin to full length or the indicated overlapping segments of SET8 fused to GST. *Top*, IB in which biotinylated molecular mass markers are visualized. *Bottom*, Ponceau staining of the same gels (shown in *grayscale*) in which standard molecular mass markers are visualized. *Asterisks*, expected positions of migration of the GST proteins.

Although, in some studies, SET8 has been reported to be solely a nuclear protein, consistent with its identified histone H4, PCNA, and UHRF1 substrates, localization of SET8 in the cytoplasm of human cells in a cell type-specific manner has also been documented previously by others (36, 37). Furthermore, even in the same cells, SET8 localization has been shown to switch between the cytoplasm and the nucleus during cell cycle progression (38). To investigate the localization of SET8 in the cytoplasm, GFP-SET8, expressed in a COS7 cell line in which it is substantially localized in the cytoplasm, was analyzed in greater detail (Fig. 1*A*). Upon screening for coassociation specifically with various cytoplasmic structural features by staining with relevant fluorescence dyes or antibodies along with GFP-SET8 expression, GFP-SET8 significantly colocalized only with α-tubulin, indicating association with MTs. MT colocalization was observed at stages throughout the cell cycle (Fig. 1*A* and Fig. S1*A*). The most obvious association was in G_1_ phase, when SET8 exhibited the same pattern as the filamentous tubulin distributed throughout the cytoplasm, emphasized by yellow in the merged image. In S phase, a larger percentage of GFP-SET8 was also nuclear (Fig. 1*A*). To verify that this colocalization with α-tubulin was not an artifact of overexpression of the fusion of SET8 with GFP or due to the use of monkey cells, immunofluorescence was used to image endogenous SET8 and α-tubulin in the HCT116 human colon cancer cell line. Again, although some SET8 was nuclear, it was abundant in the cytoplasm, where it colocalized with α-tubulin (Fig. 1*B*). In addition, we performed biochemical fractionation of human HEK293T cells (Fig. 1*C*), which were used in the subsequent experiments. Although the nuclear fraction still contained some cytoplasmic markers (tubulins, likely because of attachment of cytoplasmic proteins to the nuclear membrane), the cytoplasmic fraction lacked any significant amount of nuclear markers. The endogenous SET8 was present in the nuclear and cytoplasmic fractions, although predominantly in the nucleus in these cells (Fig. 1*C*).

The colocalization of SET8 in the cytoplasm with microtubules suggested that SET8 might be a microtubule-associated protein. Because purified tubulin preparations from mammalian tissues are known to contain microtubule-associated proteins that copurify with the polymerized tubulin, we tested whether such a preparation (>97% tubulin) contained SET8. By immunoblotting, SET8 was detectable, although as a minor component (Fig. S1*B*). To confirm that endogenous cellular SET8 associates with tubulins in HEK293T cells, we immunoprecipitated protein complexes from cell extracts. Using an antibody against SET8, α-tubulin was also precipitated. In addition, some β-tubulin coprecipitated, although to a considerably lesser extent (Fig. 1*D*). Conversely, upon expression of FLAG-tagged α-tubulin or β-tubulin in the cells, endogenous SET8 coimmunoprecipitated with both to roughly similar extents compared with the level of expression of the tagged tubulin (Fig. 1*E*). As α- and β-tubulins stably heterodimerize in cells, *in vitro* experiments were required to determine whether either of these interactions between SET8 and tubulin was direct. To this end, purified recombinant proteins fusing maltose binding protein (MBP) to either α-tubulin (TUBA1A) or β-tubulin (TUBB) were individually tested for interactions with His-tagged SET8 purified from *Escherichia coli*. SET8 interacted directly only with α-tubulin but not with β-tubulin (Fig. 1*F*). To map the region of SET8 that interacts, recombinant proteins fusing GST to full-length or the N- or C-terminal overlapping portions of human SET8 were tested for interactions *in vitro* with purified mammalian tubulin. The purified heterodimeric tubulin interacted only with the full-length and N-terminal portion of SET8, even though the C-terminal SET8 fusion protein was present at a higher level than the others (Fig. 1*G*), indicating specificity of this interaction. Taken together, these data demonstrate that α-tubulin and SET8 interact directly with each other, whereas β-tubulin only associates in a complex with SET8 in the presence of α-tubulin.

### SET8 methylates α-tubulin

**Figure 2.**
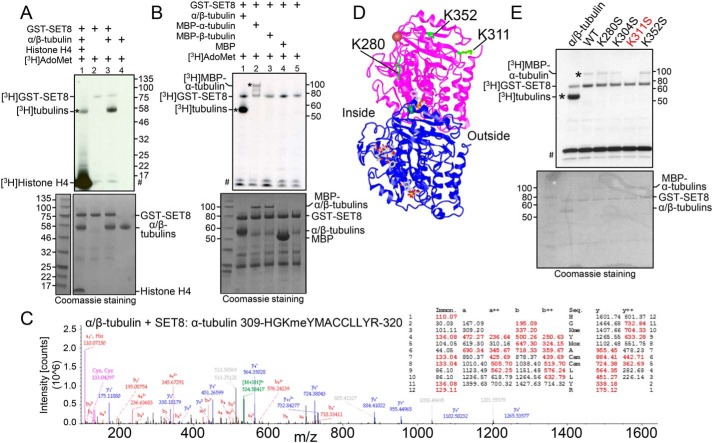
**The histone methyltransferase SET8 methylates α-tubulin at Lys^311^.** *A*, purified porcine tubulin (rPeptide, T-1201-1) is methylated by SET8. *Lane 1*, histone H4 (1 μg) was added in addition to tubulin as substrates. *Top*, autoradiogram of methyltransferase assays, showing methylation of tubulin (*asterisk*), histone H4, and automethylation of GST-SET8. #, migration of ^3^H-labeled impurities that migrated at a similar position as that of histone H4. *Bottom*, Coomassie staining of the same gel (shown in *grayscale*), indicating relative levels of the components in the reaction. *B*, recombinant human MBP–α-tubulin (*asterisk*), but not MBP–β-tubulin, is methylated by SET8. Autoradiogram (*top*) and Coomassie staining (*bottom*) are as described in *A*. Protein bands of less than 50 kDa are from the purified GST-SET8 preparation and are more evident in this experiment than in other reactions. *C*, mass spectrum and table of the expected *m*/*z* of the peptide fragments (with observed fragments in *red*), confirming methylation on Lys^311^ of α-tubulin after incubation of purified tubulin with SET8. *D*, the 3D structure of the α/β-tubulin heterodimer (PDB code 1JFF; *purple*, α-tubulin; *blue*, β-tubulin), indicating positions of lysines in α-tubulin targeted by SET8 *in vitro* (*green*). Inside and outside surfaces of the MT structure are indicated. *E*, mutation solely of Lys^311^ in recombinant MBP–α-tubulin (K311S) substantially reduced methylation by GST-SET8 *in vitro*. Autoradiogram (*top*) and Coomassie-staining (*bottom*) are as described in *A*.

SET8 was characterized historically as a histone H4K20-specific methyltransferase and subsequently as a regulator of the nonhistone protein p53. However, because SET8 bound strongly to α-tubulin, we tested whether tubulins could be a novel substrate of the enzyme. Purified porcine α/β-tubulin was incubated with the cofactor AdoMet and purified, recombinant GST-SET8. In the presence of SET8 and AdoMet, radioactivity was incorporated into a protein band migrating at the position of α- and β-tubulins in addition to less pronounced automethylation of GST-SET8 (Fig. 2*A*, *lane 3*), but no radioactive product was present at the position of α- and β-tubulins when either tubulin or SET8 was omitted from the reaction (Fig. 2*A*, *lanes 2* and *4*). Interestingly, when histone H4 was also included in the reaction, the amount of tubulin modification was reduced (Fig. 2*A*, *lane 1*), indicating that histone H4 strongly competed with tubulins for the methylation activity of SET8. Furthermore, histone H4 also competed with SET8 itself as a substrate, as shown by the significant reduction in SET8 automethylation in the presence of histone H4.

Because purified tubulin is composed of α- and β-tubulin heterodimers, we sought to determine which species is methylated by SET8. Recombinant fusion proteins of α-tubulin or β-tubulin with MBP were purified and incubated with SET8 along with the radioactive methyl donor. Upon incubation of SET8 with α/β-tubulin and AdoMet, SET8 and tubulin(s) were labeled. However, only MBP-α-tubulin, but not MBP-β-tubulin, was methylated along with SET8 itself when the individual recombinant proteins were tested (Fig. 2*B*). These data indicate that α-tubulin is the target for SET8. Mass spectrometry was used to determine which lysine residues of α-tubulin were methylated by SET8. In control samples lacking exogenous SET8, lysine methylation of α-tubulin on Lys^304^ (Fig. S2*A*) and of β-tubulin on Lys^19^ and Lys^297^ (Fig. S2*B*) were detected, none of which have been reported previously. As anticipated from the previous data (Fig. 2*B*), incubation with exogenous SET8 did not induce detectable methylation at any other sites on β-tubulin. However, SET8 did induce methylation of three additional lysine residues of α-tubulin (Lys^280^, Lys^311^, and Lys^352^) that were all monomethylated (Fig. 2*C* and Fig. S2*A*). Of these three lysines, only Lys^311^ is located on the outside surface of MTs, whereas Lys^352^ is at the interface between α-tubulin and the β-tubulin in the adjacent heterodimer and Lys^280^ is on the inside surface of MTs (Fig. 2*D* and Fig. S2, *C* and *D*). In addition, only the sequence surrounding Lys^311^ (RHGK^311^) resembles those of other known SET8 target sequences: histone H4 (RHRK^20^) and p53 (RHKK^382^) (22). In contrast, the sequences of the other α-tubulin sites, SAEK^280^ and TGFK^352^, do not resemble other known physiological SET8 targets. Therefore, to determine the relative efficiency of methylation by SET8 *in vitro* at the identified sites, each was independently mutated in the context of the full-length MBP-α-tubulin, and purified proteins were tested for incorporation of radioactivity upon incubation with SET8. Each lysine was mutated to serine, maintaining a similar structure and hydrophilicity but removing the charge. Consistent with the Lys^311^ surrounding sequence being the best match with other SET8 targets, mutation of Lys^311^ abolished modification by SET8. In contrast, mutation of Lys^280^, Lys^304^, or Lys^352^ did not appreciably affect the degree of methylation of the substrates (Fig. 2*E*).

To further test targeting of the various α-tubulin sites by SET8, peptides spanning these three sites (Lys^280^, Lys^311^, and Lys^352^) as well as Lys^40^, reported to be methylated by SETD2 (39), and Lys^304^, modified in purified porcine tubulin (Fig. S2*A*), were incubated with purified WT SET8 *in vitro*. Only the Lys^311^-containing peptide was robustly methylated (Fig. S3, *A* and *B*, and Table S1). In addition, radioactive incorporation into the Lys^311^-containing peptide was absent when incubated with catalytically inactive SET8 (D338A) *in vitro*, and methylation was abolished when the Lys^311^ residue was mutated (K311A and K311S) or already modified (monomethylated lys^311^ and acetylated lys^311^) (Fig. S3*B* and Table S1). Although the *in vitro* targeting of the α-tubulin Lys^311^-containing peptide by SET8 was robust, SET8 methylated histone H4 much more efficiently (Fig. S3*C*), consistent with the ability of histone H4 to strongly compete against tubulin for methylation by SET8 (Fig. 2*A*).

As a comparison, we tested methylation by SETD2 of the α-tubulin Lys^40^-containing peptide. The original account (39) describing methylation of α-tubulin by SETD2 included the statement in the supporting experimental procedures, as data not shown, that purified SETD2 did not methylate the target peptide *in vitro*. We confirmed the unexpected finding that methylation of the Lys^40^-containing peptide was not detectable over background despite the reported methylation of histone H3 by the purified SETD2 enzyme *in vitro* (Fig. S3*D*). Lys^40^ is the only residue in α-tubulin reported previously to be targeted by an identified tubulin methyltransferase (39). Taken together, these observations indicate that SET8 methyltransferase can directly, specifically, and effectively methylate α-tubulin at Lys^311^.

### LSF associates with SET8 and tubulin

DNA-binding proteins recruit chromatin writers to modify histones (40–43), suggesting the possibility that tubulin-binding proteins might similarly recruit SET8 to target sites on microtubules, resulting in tubulin modification. Our previous studies showed that the transcription factor LSF interacts with DNMT1, and addition of an inhibitor of the LSF–DNMT1 interaction resulted in alterations in the genomic DNA methylation profile (34); this is consistent with recruitment of DNMT1 to DNA by LSF to facilitate DNA methylation at specific sites. Because DNMT1 complexes with SET8, and both SET8 and LSF (31) are required for mitotic progression, we proposed the novel hypothesis that the transcription factor LSF might also recruit SET8 to microtubules to facilitate α-tubulin methylation by SET8. In support of this notion, there is precedent of some DNA-binding transcription factors binding microtubules (44–49) but, in all of these instances, for the purpose of sequestering the transcription factors in the cytoplasm and/or facilitating their transport into the nucleus.

**Figure 3.**
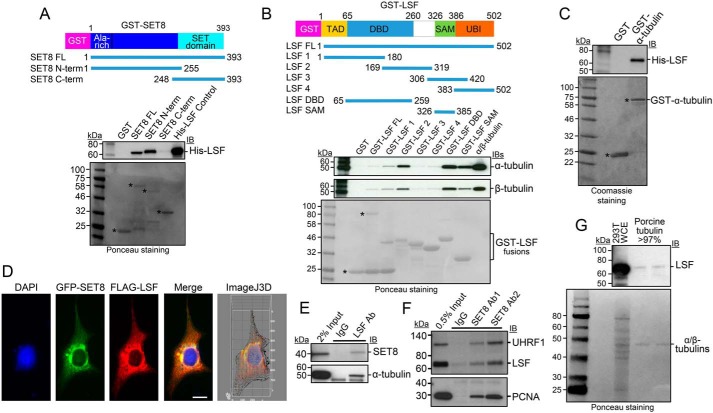
**LSF interacts directly with SET8 and tubulin.** *A*, GST pulldown analysis of purified His-LSF to full length or the indicated overlapping segments of SET8 fused to GST. *Top*, IB in which biotinylated molecular mass markers are visualized. *Bottom*, Ponceau staining of the same gels (shown in *grayscale*) in which standard molecular mass markers are visualized. *Asterisks*, expected positions of migration of the GST proteins. *B*, GST pulldown analysis of purified porcine tubulin to purified full length or the indicated overlapping segments of LSF fused to GST. Gels are as described in *A. Asterisks* and *bracket*, expected positions of migration of the GST proteins. *C*, GST pulldown analysis of recombinant, purified His-tagged LSF to purified α-tubulin fused to GST. Gels are as described in *A*, except that the protein gel was stained with Coomassie. *D*, plasmids expressing 3×FLAG-LSF and GFP-SET8 were transfected into COS7 cells. Anti-FLAG antibody was visualized with a red fluorescent secondary antibody, and DNA was visualized with DAPI. The merged image indicates colocalization of GFP-SET8 with FLAG-LSF (*yellow*), concentrated largely near the nuclear membrane (Manders correlation coefficient of LSF and SET8 colocalization is 0.9, as determined via ImageJ 3D analysis). The majority of overexpressed 3×FLAG-LSF was cytoplasmic, with only a minority detected in the nucleus. *E*, specific coimmunoprecipitation of endogenous SET8 (*top*) and endogenous α-tubulin (*bottom*) from HEK293 cellular extracts, using antibodies to LSF compared with control IgG. *F*, specific coimmunoprecipitation of endogenous LSF from HEK293 cellular extracts using antibodies to SET8 (Ab1, Active Motif; Ab2, Millipore) compared with control IgG. Coimmunoprecipitation of PCNA and UHRF1 is also shown as a positive control. *G*, immunoblotting of purified porcine brain tubulin (rPeptide, >97%) shows the presence of LSF using an LSF mAb; representative also of results obtained using a separate source of purified tubulin: MP Biomedicals, more than 99%. Positive control for LSF migration, HEK293T whole-cell extract (*293T WCE*). *Top*, immunoblot. *Bottom*, Ponceau staining using standard molecular mass markers.

To test our hypothesis, multiple assays were initially performed to evaluate whether LSF interacts with SET8 and tubulin(s). *In vitro*, direct interaction between recombinant, purified SET8 and purified LSF was evaluated by a GST pulldown assay (Fig. 3*A*). Using fusion proteins between GST and full-length SET8 or its N- or C-terminal fragments, His-LSF bound specifically to the N-terminal region of SET8 (Fig. 3*A*), the same domain that bound purified tubulins (Fig. 1*F*). Binding of purified α/β-tubulin to purified GST-LSF was also evaluated and mapped to specific regions within LSF. Both α- and β-tubulins showed binding profiles similar to the panel of LSF fusion proteins, as expected, given their stable heterodimeric structure (Fig. 3*B*). Binding of tubulins to the full-length GST-LSF was greater than to the control GST, although quite weak compared with some of the other fusion proteins; this was ascribed to the sensitivity of the full-length GST-LSF fusion protein to cleavage in bacterial culture, resulting in a significant fraction of the purified preparation representing the GST domain alone. However, the tubulins interacted strongly with two specific domains of LSF whose GST fusion proteins were stable: the DNA-binding domain and, to a lesser extent, the sterile α motif domain (Fig. 3*B*). Further analysis suggests that it is the C-terminal portion of the DNA-binding domain that contains the tubulin interaction surface in this domain because the GST-LSF 2 protein also binds both tubulins to a high degree. Finally, purified His-LSF also interacted in parallel assays with purified recombinant full-length GST–α-tubulin (Fig. 3*C*). These *in vitro* protein–protein interaction results indicate that all pairwise interactions among SET8, LSF, and α-tubulin occur through direct binding with each other.

To examine whether interactions of LSF with both SET8 and tubulin also take place in cells, multiple approaches were taken. First, upon coexpression of GFP-SET8 and 3×FLAG-tagged LSF in transient transfection assays, the two proteins significantly colocalized, predominantly in the cytoplasm (Fig. 3*D*). Although LSF, as a transcription factor, is localized in the nucleus, endogenous LSF has also been shown to localize in the cytoplasm in a cell type–specific manner (36), consistent with these immunofluorescence results. Second, the presence of complexes between endogenous cellular proteins was tested by coimmunoprecipitation experiments using HEK293T cell lysates. With antibodies against endogenous LSF, but not control antibodies, both endogenous SET8 and endogenous α-tubulin coimmunoprecipitated with LSF (Fig. 3*E*). Reciprocally, SET8 antibodies not only specifically coimmunoprecipitated its previously identified partner proteins, PCNA (50–52) and UHRF1 (37), but also endogenous LSF (Fig. 3*F*). Finally, the possibility of relevant LSF–tubulin interactions was investigated by analyzing whether LSF was present in commercial, highly purified tubulin preparations. These preparations are obtained in part by multiple rounds of polymerization/depolymerization of tubulin and are more than 97%–99% pure. They are well known to contain additional proteins that are defined as MAPs. Immunoblots using an LSF mAb did indeed detect a band comigrating with LSF, albeit at a very low level (Fig. 3*G*). LSF was reproducibly detected in this manner in multiple commercially purified preparations of tubulin.

Taken together, these results demonstrate that LSF interacts directly with both SET8 and α-tubulin *in vitro* and also associates with both of these proteins *in vivo*. Furthermore, LSF, although a transcription factor, appears to be a previously unidentified MAP.

### LSF promotes tubulin methylation by SET8

**Figure 4.**
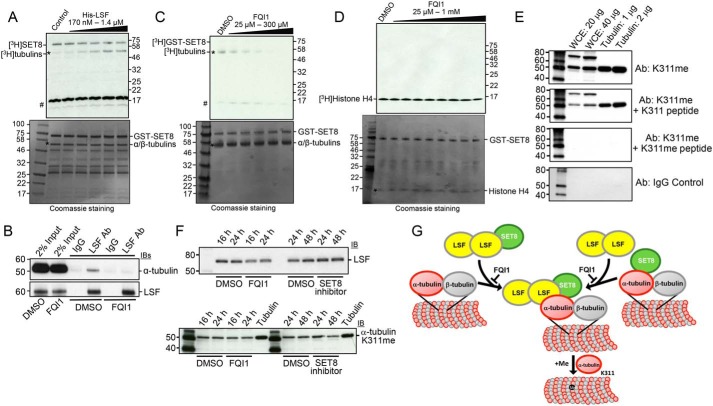
**LSF and FQI1 oppositely affect methylation of tubulin by SET8.** *A*, tubulin (>99%, MP Biomedicals) methylation reactions were performed with addition of the indicated increasing range of concentrations of LSF. *Top*, autoradiogram of methyltransferase assays, showing methylation of tubulin (*asterisk*) and automethylation of GST-SET8. The higher relative levels of GST-SET8 to α/β-tubulin in this experiment led to greater initial automethylation of SET8 relative to tubulin methylation. #, migration of ^3^H-labeled impurities. *Bottom*, Coomassie staining of the same gel (shown in *grayscale*), indicating relative levels of the components in the reaction. As in Fig. 2*B*, protein bands of less than 50 kDa are from the purified GST-SET8 preparation and are more evident in this experiment. *B*, coimmunoprecipitation of endogenous α-tubulin with endogenous LSF from HEK293T cell lysates was disrupted upon treatment of the cells with 2.5 μm FQI1 for 24 h. *C*, tubulin (>99%, MP Biomedicals) methylation reactions were performed with addition of the indicated increasing range of concentrations of FQI1. At 100 μm FQI1 (*lane 4*), methylation is decreased ∼3-fold. Gels are labeled as in *A. D*, histone H4 methylation reactions at limiting amounts of histone H4 (200 ng) were performed with addition of the indicated increasing range of concentrations of FQI1. Gels are labeled as in *A. E*, specific methylation of tubulin on Lys^311^. Immunoblots of HEK293T cell lysates and purified tubulin, at the indicated concentrations, with α-tubulin K311me or nonspecific IgG antibodies. Specificity to methylated Lys^311^ was demonstrated by preincubation of the antibody with methylated *versus* nonmethylated α-tubulin Lys^311^ peptides. *F*, treatment of HEK293T cells with either LSF or SET8 inhibitors somewhat reduces the level of methylated Lys^311^ on α-tubulin. *G*, model of recruitment and/or activation of SET8 at microtubules by LSF and subsequent methylation of α-tubulin by SET8.

The demonstration of pairwise, physical interactions between LSF, tubulin, and SET8 set the stage for directly testing the hypothesis that LSF could mediate the methylation of α-tubulin by SET8. Thus, recombinant GST-SET8 and the radioactive methyl donor were incubated with tubulin in the presence of increasing concentrations of purified His-LSF (Fig. 4*A*). Tubulin methylation increased upon increasing LSF from a 1:4 to 2:1 molar ratio of LSF:GST-SET8, suggesting that LSF can mediate tubulin methylation by SET8. Note that, in this experiment, there was more SET8 relative to tubulin than in other experiments (Fig. 4*A*, *bottom*), resulting in a greater degree of automethylation of SET8 compared with tubulin methylation (Fig. 4*A*, *top*), presumably because of substrate competition between SET8 itself and α-tubulin. A similar experiment was performed using recombinant MBP–α-tubulin as substrate for SET8, which also showed that increasing levels of LSF enhanced methylation of MBP–α-tubulin (Fig. S4*A*).

The LSF small-molecule inhibitor FQI1 inhibits LSF binding to DNA (35), as well as binding of LSF to certain protein partners (34). To determine whether FQI1 would diminish the interaction between LSF and α-tubulin in cells, cell lysates from vehicle- *versus* FQI1-treated cells were analyzed by coimmunoprecipitation assays. These demonstrated a significant reduction in the LSF-α-tubulin interaction after FQI1 incubation for 24 h (Fig. 4*B*). Because FQI1 can inhibit the LSF–tubulin interaction *in vivo*, it was used to interrogate whether the interaction of LSF and tubulin was important for stimulating SET8-mediated methylation of α-tubulin *in vitro*. Given that LSF is already present in the tubulin preparations, FQI1 was initially added to reactions containing only SET8, a radioactive methyl donor, and purified tubulin. Tubulin methylation decreased with increasing concentrations of FQI1 (Fig. 4*C*), consistent with the presence of LSF and its ability to enhance SET8-dependent tubulin methylation. Whether FQI1 specifically inhibits LSF in these assays was tested in two ways. First, it was demonstrated that the presence of FQI1 prevented any increase in tubulin methylation upon addition of purified His-LSF (Fig. S4*B*, compare *lanes 7* and *8*). Second, the possibility that FQI1 directly inhibits SET8 catalytic activity was tested using histone H4 instead of α-tubulin as a substrate. Limiting amounts of histone H4 were added in this experiment to enhance the sensitivity of the assay. FQI1 did not inhibit methylation of histone H4 by SET8 (Fig. 4*D*), in contrast to its effect on α-tubulin methylation (Fig. 4*C*). In addition, when SET8 was incubated with histone H4 plus whole-cell extract in the presence of the radioactive methyl donor, FQI1 did not appreciably diminish methylation of any other proteins in the extract (Fig. S4*B*, compare *lanes 1* and *2*), in contrast to its ability to inhibit methylation of tubulin (Fig. S4*B*, compare *lanes 3* and *4*).

To verify that this methylation occurs *in vivo*, we generated an antibody against an α-tubulin peptide containing K311me. Upon immunoblotting HEK293T whole-cell lysates, only two proteins were identified with the antibody, one of which comigrated with tubulins (Fig. 4*E*). Purified mammalian tubulin was also detected when sufficiently large amounts were loaded onto the gel. Specificity of the interaction to the methylated lysine in both proteins detected was demonstrated by the ability to compete the signal with methylated but not unmethylated peptide. The identity of the larger protein is not yet known and the subject of ongoing investigation, but this experiment definitively demonstrates that α-tubulin is methylated on Lys^311^ *in vivo*. Upon treating cells with a SET8 inhibitor, the degree of methylation of α-tubulin, as detected with the K311me-specific antibody, was diminished (Fig. 4*F*, *bottom*). Treatment with FQI1 to inhibit LSF reduced the level of LSF in these HEK293T cells (Fig. 4*F*, *top*), as noted previously (35). More importantly, FQI1 also partially diminished the degree of Lys^311^ methylation of α-tubulin, but to a lesser degree than the SET8 inhibitor (Fig. 4*F*, *bottom*).

To probe whether LSF recruits SET8 to tubulin, we used FQI1 to disrupt the LSF–tubulin interactions (Fig. 4*B*). FQI1 did diminish coimmunoprecipitation of endogenous tubulin with SET8 antibodies (Fig. S4*C*), supporting the recruitment model. However, a caveat to this straightforward interpretation was that SET8 immunoprecipitation was also somewhat diminished, although to a lesser degree, after incubation of the cells with FQI1.

Taken together, these findings indicate that SET8 methylates α-tubulin at Lys^311^. Furthermore, they suggest that LSF enhances this ability of SET8 to modify α-tubulin and, conversely, that FQI1 therefore impedes methylation of α-tubulin by SET8. The data support a model in which LSF recruits SET8 to tubulin and/or in which LSF binding as a ternary complex with both SET8 and tubulin activates the methylase activity of SET8 already associated with tubulin (Fig. 4*G*).

## Discussion

Tubulin PTMs are generally thought to regulate binding of proteins to the microtubule cytoskeleton, thereby regulating microtubule function, the cell cycle, and signaling events in the cell. To date, a large variety of MAPs have been characterized that stabilize or destabilize microtubules, are associated with coupling of molecular motors and microtubules, and play critical roles in spindle formation (53). Here we identify additional sites of methylation on tubulins - in particular methylation of Lys^311^ on α-tubulin, we identify two additional unanticipated MAPs - the *N*-lysine methyltransferase SET8, which methylates α-tubulin Lys^311^, and the transcription factor LSF, and we provide support for a novel mechanism for facilitating tubulin modifications.

### Tubulin methylases and sites of methylation

A number of posttranslational modifications of microtubules are well established, although limited insights have been obtained regarding their biological roles, as tubulin PTMs have generally remained less amenable to straightforward functional studies. Identified posttranslational modifications of tubulins mediated by specific modifying enzymes include acetylation of lysine 40 in α-tubulin by αTAT1 (MEC-17 in *Caenorhabditis elegans*); deacetylation of the same residue by Sirt2 (Sirtuin type2), HDAC5, and HDAC6; and polyglutamylation of both α- and β-tubulins at multiple C-terminal glutamate residues by TTLL4, TTLL5, and TTLL7 (54, 55). Despite extensive research regarding these various modified sites and their relevant enzymes, only one study previously identified lysine methylation of a tubulin (39), which is the focus of this report. Walker and co-workers (39) reported that SETD2, known as a histone methyltransferase for a chromatin activation mark, trimethylated histone H3 on lys^36^, also methylates α-tubulin at Lys^40^. Surprisingly, however, although purified SETD2 (aa 1392–2564) apparently methylated purified bovine tubulin and recombinant TUBA1A-myc *in vitro*, it was noted, as data not shown in the report, that the enzyme was not able to methylate a Lys^40^-containing peptide or purified GST-TUBA4A (see supporting experimental procedures in Ref. 39). This is despite the fact that all six TUBA1 and TUBA3 isoforms have identical amino acid sequences between residues 1–74, and TUBA4A differs at only four residues in that stretch, with the nearest nonidentical residue 10 away from Lys^40^ (residues 7, 16, 50, and 54). We now confirmed the unexpected finding that purified SETD2 (aa 1392–2564) did not detectably methylate a α-tubulin Lys^40^-containing peptide *in vitro*. It will be interesting to understand the basis of this discrepancy.

Here we describe a distinct, novel lysine methylation of α-tubulin at Lys^311^ and identify an enzyme responsible for its modification both *in vitro* and *in vivo* as SET8, which is fully capable of methylating the target peptide as well as intact recombinant human protein and purified porcine tubulin. Given that the SET8 inhibitor did not entirely eliminate the K311me modification in cells, it is possible that another, still unidentified methyltransferase also contributes to modification of this lysine. The RHGK^311^ motif is highly conserved in α-tubulins, being present in eight human TUBA isotypes (TUBA1A–TUBA1C, TUBA3C–TUBA3E, TUBA4A, and TUBA8).

In addition, we identify methylation of β-tubulin purified from mammalian brain at Lys^19^ and Lys^297^. Lys^19^ is conserved in all human β-tubulin isotypes, and the surrounding sequence in six of the nine isotypes. Lys^297^ is conserved in seven of nine β-tubulin isotypes, whereas two isotypes instead have Arg^297^. We are pursuing identification of the responsible enzymes.

### Biological consequences of tubulin methylation

SETD2 and SET8 not only target different sites in α-tubulin but lead to differing methylation states (tri- *versus* monomethylation, respectively). Thus, it is anticipated that each would lead to distinct biological consequences, including binding of different proteins. This is especially the case because these sites are on completely different locations on the microtubules: Lys^40^ in the lumen and Lys^311^ on the outer surface. Phenotypically, disruption of SETD2 or SET8 results in mitotic defects and subsequent genomic instability but with distinct features. SETD2-null mouse embryonic fibroblasts exhibit mitotic delay with delayed congression, multipolar spindles, lagging chromosomes, and cytokinesis failure, resulting in polyploidy and polynucleation (39). In contrast, loss of SET8 results in premature chromosome condensation, leading to delayed mitotic progression in addition to defects in S phase (11–19). Conversely, lack of timely SET8 degradation in mitosis also delays progression between metaphase and anaphase (20, 21).

Precise modulation of SET8 levels is required for proper mammalian cell cycle progression. Previous reports have suggested that SET8 and its modification of histone H4, H4K20me1, function as novel regulators of cell cycle progression, with a focus on regulation of S phase (56). With our demonstration that SET8 can also methylate α-tubulin, the roles of nonhistone substrates must also be considered as causes for SET8-mediated regulation of the cell cycle, and in particular of mitosis, when SET8 is most abundant (9). Similarly to mammals, SET8 is an essential protein for *Drosophila melanogaster*, as SET8 mutants die during larval development. However, flies in which all histone H4 genes were replaced with multiple copies of the mutant histone H4K20A unexpectedly survived to adulthood without apparent phenotypic defects, although they did exhibit a significant delay in development (57). Thus, contrary to the prevailing view, histone H4 was not the most critical biological target for SET8. Given the minimal biological effects of mutating histone H4K20 in *Drosophila*, we propose that α-tubulin methylation is a strong candidate for mediating critical SET8 consequences. Notably, *D. melanogaster* and human α-tubulins are 98% identical with all lysines throughout the sequence being conserved.

### Targeting SET8 to tubulin by transcription factor LSF: a general model

Beyond identification of the tubulin PTMs and the enzymes that catalyze these modifications is the question of what drives the spatiotemporal access of such enzymes to MTs. Relevant to this process, we also demonstrate here that a transcription factor, LSF, apparently moonlights as a microtubule-associated protein and that LSF has the ability to recruit SET8 to tubulin and/or enhance SET8's enzymatic modification of α-tubulin. Such a recruitment mechanism would mirror mechanisms of targeting histone writers to chromatin, expanding the model of the parallel nature between the generation of the histone and tubulin codes. Furthermore, these data suggest that transcription factors more generally may be able to regulate tubulin modifications and, thereby, microtubule dynamics. Although several transcription factors have been reported previously to bind microtubules, including MYC (44, 45), MIZ-1 (46), p53 (47, 49), and SMADs (48), in all of these cases, the biological relevance proposed or demonstrated was to sequester the transcription factors in the cytoplasm and/or to help transport the transcription factor into the nucleus. Thus, all previous transcription factor–microtubule interactions were proposed to regulate transcription activity, not microtubule function. The *in vitro* results regarding LSF, although requiring further validation *in vivo*, provide the first instance in which binding of a transcription factor directly to microtubules affects tubulin modification and, presumably, to altered microtubule function. We propose that this may represent a new paradigm that reflects functions of other transcription factors as well.

### Relevance of LSF and SET8 to cancer

LSF, like SET8, is required for mitotic progression, as evidenced by mitotic defects upon reduction of LSF by siRNA or by inhibition of LSF activity by the small-molecule inhibitor FQI1 (31, 33). Consistent with LSF's role in cell cycle progression (30, 31, 33), LSF has been implicated as an oncogene in multiple cancer types (29). In particular, LSF enhances tumorigenesis in hepatocellular carcinoma (23, 26). Hepatocellular carcinoma is the sixth most common cancer worldwide and the second highest cause of cancer-related death globally (58). LSF is overexpressed in human hepatocellular carcinoma cell lines and over 90% of human hepatocellular carcinoma patient samples, showing significant correlation with stages and grades of the disease (23), and elevated levels of LSF in patient tumors correlate with decreased survival (28). SET8 levels are also elevated and contribute, by multiple mechanisms, to cancer progression (56, 59), including in hepatocellular carcinoma (60). Furthermore, elevated expression of specific α-tubulins (*e.g.* TUBA1B) has also been associated with this disease (61).

Mitosis, in which both LSF and SET8 are involved, is viewed as a vulnerable target for inhibition in cancer (62). The lead LSF inhibitor, FQI1, induces apoptosis in hepatocellular carcinoma cell lines *in vitro* as a consequence of mitotic defects and significantly inhibits tumor growth in multiple mouse hepatocellular carcinoma models, with no observable toxicity to normal tissues (31, 35). Our new findings that LSF interacts with α-tubulin and SET8 and that FQI1 hinders the LSF-α-tubulin interaction may relate to the impact of LSF inhibitors in hepatocellular carcinoma cells and tumors. Given the current large unmet medical need, further investigation into the relevance of the LSF–α-tubulin–SET8 pathway for hepatocellular carcinoma and other cancer types in which LSF is oncogenic may aid novel targeted and effective treatments.

## Experimental procedures

### Cell culture, immunoprecipitation, and immunofluorescence

HEK293T and COS7 cells were cultured in DMEM supplemented with 10% fetal bovine serum at 37 °C. HCT116 cells were cultured in McCoy's medium with 10% fetal bovine serum according to ATCC recommendations. For treatment with LSF- and SET8-specific inhibitors, HEK293T cells were incubated with 2.5 μm FQI1 (Millipore/Sigma, 438210) or 10 μm UNC0379 (Selleckchem, S7570), respectively. FQI1 treatment was for 24 h or as indicated.

Immunoprecipitation and immunofluorescence experiments were carried out as described previously (63, 64). For the immunoprecipitation, 1 mg of total HEK293T cellular extract was incubated with 5 μg of anti-SET8 antibody (Active Motif, 61009), anti-SET8 antibody (Millipore, 06-1304), anti-LSF antibody (Millipore, 17-10252), or mouse anti-FLAG antibody (Sigma-Aldrich, F1804,). The immunoprecipitates were blotted with anti-α-tubulin (Sigma, T9026), anti-β-tubulin (Sigma, T8328), anti-SET8 (Abcam, ab3798), anti-Pr-Set7 (D11, Santa Cruz, sc-377034), anti-LSF (BD Biosciences, 610818), anti-PCNA (Cell Signaling Technology, 2586), anti-UHRF1 (anti-ICBP90, BD Biosciences, 612264), or anti-FLAG (Cell Signaling Technology, 14793) antibodies according to the manufacturer's dilution recommendations. Cellular extracts were also immunoprecipitated with normal IgG (Cell Signaling Technology) as a negative control for all immunoprecipitation experiments.

For immunofluorescence to detect α-tubulin and SET8 colocalization, COS7 cells were grown on coverslips and transfected with a GFP-SET8 expression plasmid. After cells were fixed with paraformaldehyde, they were incubated with anti-α-tubulin, and the microtubules were visualized with an anti-mouse IgG coupled with Alexa Fluor 488 (Molecular Probes) using a confocal microscope (Zeiss LSM510). For detection of endogenous α-tubulin and SET8 colocalization, HCT116 cells were fixed with formaldehyde followed by methanol and blocked for 1 h with 5% bovine serum albumin in PBS with 0.1% Tween 20. Cells were incubated with anti-SET8 antibody (Millipore), followed by anti-rabbit IgG coupled with Alexa Fluor 488 and anti-α-tubulin (Sigma, T9026) and then anti-mouse IgG coupled with Alexa Fluor 594 (Molecular Probes). Colocalization was detected using an LSM880 confocal microscope (Zeiss). For detection of SET8 and LSF colocalization, COS7 cells were cotransfected with GFP-SET8 and 3×FLAG-LSF expression plasmids; the epitope-tagged LSF was detected by mouse anti-FLAG antibody (F3165, Sigma-Aldrich) and visualized with an anti-mouse IgG coupled with Alexa Fluor 488 (Molecular Probes). DAPI was used to stain nuclear DNA.

### GST and MBP pulldown assays

LSF, SET8, and α-tubulin complementary DNAs were cloned into the pGEX-5X-1 (GE Healthcare) or pMalC4X (New England Biolabs) vector, and GST-tagged or MBP-tagged proteins were captured using GSH-Sepharose beads (GE Healthcare) or amylose resin (New England Biolabs), respectively. Sepharose beads containing ∼10 μg of fusion protein were incubated for 2 h at 4 °C with purified tubulin (MP Biomedicals), recombinant His-tagged LSF, or recombinant His-tagged SET8, the latter two purified from *E. coli*. Proteins bound to the beads were resolved by 10%–20% SDS-PAGE. LSF, SET8, and α-tubulin were visualized by immunoblotting using anti-LSF (BD Biosciences), anti-SET8 (Active Motif), or anti-α-tubulin (Sigma-Aldrich), respectively.

### In vitro methylation assays

Approximately 1 μg of recombinant GST-SET8 (in 50% glycerol) and 2 μg of purified tubulin (MP Biomedicals, 08771151), recombinant MBP-α-tubulin, or recombinant MBP-β-tubulin were incubated with 6 μm radioactively labeled [^3^H]AdoMet (PerkinElmer Life Sciences, NET155V001MC) in 5 mm Tris (pH 8.0) and 0.5 mm DTT at room temperature overnight. As indicated, histone H4 (New England Biolabs, M2504S), recombinant His-LSF protein, or FQI1 inhibitor was added to the reaction. Samples were separated by electrophoresis through a 10% *N*-[2-hydroxy-1,1-bis(hydroxymethyl)ethyl]glycine gel (Invitrogen), and the gel was stained with Coomassie Brilliant Blue (shown in *grayscale* in the figures) and incubated with EN3HANCE (PerkinElmer Life Sciences) solution. The gel was dried and exposed to autoradiography film for 1 week. For the peptide assays, the specific peptides of α-tubulin were synthesized from AnaSpec Inc. Sequences are listed in Table S1. 2 μg of each peptide and 2 μg of purified WT or mutant SET8 or SETD2 (aa 1392–2564, Active Motif) were incubated with radioactively labeled [^3^H]AdoMet at room temperature overnight. Samples were spotted onto P81 filters (Whatman, 3698325), and the filters were washed three times with 0.3 m ammonium bicarbonate. The level of incorporated [^3^H]CH_3_ was determined using liquid scintillation counting.

### Mass spectrometry analysis

For identification of tubulin modifications, purified tubulin (MP Biomedicals, 08771151) was incubated with nonradioactive AdoMet with or without recombinant GST-SET8 overnight at room temperature, and the samples were separated by electrophoresis through a 10% Tris-glycine gel. Excised gel bands were digested with either subtilisin or trypsin in 0.01% ProteaseMax in 50 mm NH_4_HCO_3_ for 1 h at 50 °C. Digestion was quenched with TFA, and samples were dried. Each digest was individually reconstituted and analyzed by direct injection onto an analytical column 25 cm 100 μm ID Aqua 3 μm with Easy n1000 nLC-QExactive at 300 nL/min. Acquired higher energy collision dissociation spectra were searched using Proteome Discoverer 2.0.0.802 with Sequest using the SWISSPROT June 2015 database (416,061 sequences) and Cys = 57.02146 static modification. Dynamic modifications were set for Met = 15.99492. Two missed and/or nonspecific cleavages were permitted. Searches were semispecific (trypsin semispecific R,K) with K = 14.016 dynamic modification. The mass tolerance for precursor ions was 10 ppm and for fragment ions 0.02 Da. Results were filtered with Percolator (q < 0.01) for high-confidence spectrum matches. The target strict false discovery rate was 0.01, as determined by Percolator.

### Biochemical fractionation of HEK293T cells

Subcellular fractions from cultured HEK293T cells were obtained using a cell fractionation kit (Cell Signaling Technology, 9038) according to the manufacturer's protocol. Cytoplasm, nuclear, and whole-cell extracts were separated by electrophoresis through 10%–20% Tris-glycine, and the resulting membrane was immunoblotted with anti-MEK1/2 (Cell Signaling Technology, 8727), anti-histone H3 (Cell Signaling Technology, 9715), anti-α-tubulin (Sigma, T9026), anti-β-tubulin (Abcam, ab15568), and anti-SET8 (Active Motif, 61009).

### K311me–α-tubulin antibody

The custom rabbit polyclonal antibody was generated and purified by Eurogentec using the peptide CDPRHK(me)YMA. Specific antibodies were purified using a methylated peptide-conjugated resin followed by depletion of unmethylated peptide reactivity. Quality control ELISA analysis indicated specificity to the methylated peptide. Immunoblotting with the antibody was performed with 2 μg of purified antibody; for the indicated blots, the antibody was preincubated overnight with 100 μg of either methylated or unmethylated peptide prior to incubation with the membranes.

### Data Availability

The tubulin methylation MS data from this publication, entitled Microtubule methylation LC-MSMS, have been deposited to the ProteomeXchange Consortium via the PRIDE (65) partner repository and assigned the dataset identifier PXD014257.

## Author contributions

H. G. C. conceptualization; H. G. C., P.-O. E., and C. R. data curation; H. G. C. formal analysis; H. G. C. validation; H. G. C., P.-O. E., and C. R. investigation; H. G. C., P.-O. E., C. R., J. L., and S. E. S. methodology; H. G. C. and U. H. writing-original draft; H. G. C., S. P., and U. H. writing-review and editing; P.-O. E., C. R., and U. H. visualization; S. P. resources; S. P. and U. H. supervision; S. P. funding acquisition; S. P. and U. H. project administration.

## Supplementary Material

Supporting Information

## Footnotes

4 The abbreviations used are:

MT: microtubule
PTM: posttranslational modification
MAP: microtubule-associated protein
H4K20me1: monomethylated histone H4 on lys^20^
LSF: late SV40 factor
PCNA: proliferating cell nuclear antigen
MBP: maltose binding protein
K311me: monomethylated lys^311^
aa: amino acids
Ab: antibody
IB: immunoblot
DAPI: 4′,6-diamidino-2-phenylindole.
