# Supplementary material for: The microtubule-associated histone methyltransferase SET8, facilitated by transcription factor LSF, methylates α-tubulin
Source: J Biol Chem. 2020 Feb 28;295(14):4748–59. doi: 10.1074/jbc.RA119.010951 (PMC7135998; doi:10.1074/jbc.RA119.010951)
Supplement: Supporting Information [file supp_295_14_4748__index.html]

The microtubule-associated histone methyltransferase SET8, facilitated by transcription factor LSF, methylates α-tubulin — SET8-mediated methylation of α-tubulin on K311 — The microtubule-associated histone methyltransferase SET8, facilitated by transcription factor LSF, methylates α-tubulin — SET8-mediated methylation of α-tubulin on Lys311 — Supporting Information 

# The microtubule-associated histone methyltransferase SET8, facilitated by transcription factor LSF, methylates α-tubulin

## Supporting Information

- Supporting Information - Supplementary Table S1 and Figures S1-S4
